# Relation between carotid intima media thickness and oxidative stress markers in type 1 diabetic children and adolescents

**DOI:** 10.1186/2251-6581-12-50

**Published:** 2013-12-19

**Authors:** Mona H El Samahy, Randa M Matter, Omneya I Youssef, Manal A Shams El Din El Telbany, Nermeen A Kamal

**Affiliations:** 1 Departments of Pediatrics, Faculty of Medicine, Ain Shams University, 29dar el ezz, Medinet el Zahraam, Helmeyet el Zaytoon, Cairo, Egypt; 2 Clinical Pathology, Faculty of Medicine, Ain Shams University, Cairo, Egypt

**Keywords:** Nitric Oxide, Total Antioxidant Capacity, Coronary Artery Disease Patient, Carotid Intima Media Thickness, Diabetes Duration

## Abstract

**Background:**

Carotid intima media thickness (CIMT) is a non invasive marker of subclinical atherosclerosis. Hyperglycemia, oxidatively modified atherogenic lipoproteins and advanced glycation end products are linked to increased oxidative stress in diabetes. We aimed to find out the relation between carotid intima media thickness in type 1 diabetic children and adolescents and plasma nitric oxide and total antioxidant capacity levels as markers of oxidative stress.

**Methods:**

This study included 50 children and adolescents with type 1 diabetes mellitus with mean age (9.7 ± 3.4 years) and 50 healthy age and sex matched controls. They were subjected to assessment of hemoglobin A1c, total cholesterol and triglycerides, serum total antioxidant capacity, serum nitric oxide (NO) by colorimetric method and carotid intima media thickness by B-mode ultrasound.

**Results:**

There was significant elevation in serum nitric oxide (17.07 ± 6.4 vs 12.6 ± 4.7 μmol/L; p < 0.001), CIMT (0.47 ± 0.04 vs 0.39 ± 0.02 mm; p < 0.001) and significant reduction in serum total antioxidant capacity (0.41 ± 0.29 vs 0.87 ± 0.23 mmol/L; p < 0.001) in diabetic patients compared to controls. Carotid intima media thickness was correlated positively with nitric oxide (r = 0.402, p = 0.01) and negatively with total antioxidant capacity (r = -0.341, p = 0.02). Carotid intima media thickness was also correlated positively with age, duration of diabetes but not correlated with glycemic control or lipid profile.

**Conclusion:**

The significant elevation in nitric oxide and reduction in total antioxidant capacity in children and adolescents with type 1 diabetes mellitus with their correlation with carotid intima media thickness may reflect the role of oxidative stress in the development of atherosclerosis in young type 1 diabetic subjects.

**Electronic supplementary material:**

The online version of this article (doi:10.1186/2251-6581-12-50) contains supplementary material, which is available to authorized users.

## Introduction

The atherogenicity of type 1 diabetes has been increasingly recognized [1]. Patients with diabetes show a 2- to 10-fold risk for developing atherosclerotic lesions compared with the normal population [2, 3]. Even if these complications become manifest in the adult diabetic patient, the process of vascular changes starts much earlier [4, 5]. The most significant changes in early subclinical period of atherosclerotic disease are endothelial dysfunction and increase in intima-media thickness observed in all arterial beds [6]. Common carotid artery intima-media thickness (CIMT), measured by high-resolution B-mode ultrasonography, is a noninvasive marker of subclinical atherosclerosis [7–9]. Normative values for CIMT are available for subjects aged 10 to 20 years [10, 11].

Mechanisms involved in the increased oxidative stress in diabetes include not only oxygen free radical generation due to nonenzymatic glycosylation (glycation), autooxidation of glycation products, but also changes in the tissue content and activity of antioxidant defense systems. Increased levels of the products of oxidative damage to lipids have been detected in serum of diabetic patients, and their presence correlates with the development of complications [12–18].

Oxidative stress plays a pivotal role in the development of diabetes microvascular and cardiovascular complications [19].

In the diabetic macrovascular and in the heart, this appears to be a consequence of increased oxidation of fatty acids, resulting in part from pathway-specific insulin resistance [19].

Nitric oxide has cellular antioxidant and pro-oxidant actions [20]. The endothelial nitric oxide (NO) system plays a pivotal role in vascular physiology and pathology. NO is a potent vasodilator agent with anti-hypertensive, anti-thrombotic, anti-atherogenic, and anti-smooth muscle proliferative properties [21]. However, high amounts of NO produced by inducible NO synthase (iNOS) and/or peroxynitrite (ONOO-), a reactive intermediate of NO with superoxide anion are involved in pro-inflammatory reactions and tissue damage as well [22].

Total Antioxidant Capacity (TAC) is capable of serving as a parameter to monitor diabetes in patients with type 1 DM [23]. A depletion of the total antioxidant capacity is associated with a higher incidence of diabetic complications [24].

The relation between oxidative stress markers and carotid intima media thickness in type 1 diabetic children and adolescents was not extensively studied. Hence, this study aimed at assessment of carotid intima media thickness in children and adolescents with type1 diabetes mellitus in relation to plasma nitric oxide and plasma total antioxidant capacity levels and with diabetes duration, glycemic control and microvascular complications.

## Patients and methods

This case control study was conducted at the Pediatric Diabetes Clinic, Children’s Hospital, Ain Shams University, Cairo, Egypt in the period from April 2011 to February 2012.It included 50 patients with type 1 diabetes mellitus(DM) regularly attending the clinic. They were 37 females (74%) and 13 males (36%). Their ages ranged from 6–16 years with a mean age of 9.7 ± 3.4 years. Their duration of illness ranged from 1–13 years with mean diabetes duration of 4.5 ± 3.5 years. Patients were further subdivided into two groups according to the duration of diabetes: (Group I) included 22 children and adolescents with diabetes duration of 5 years or more, (Group II) included 28 children and adolescents with diabetes duration of less than 5 years. Fifty age and sex matched healthy individuals were included as a control group. They were 14 males and 36 females. Their age ranged from 2–14 years with mean age of 9.8 ± 3.14 years.

The study was approved by the Ethical Committee of Ain Shams University Faculty of Medicine.

### Inclusion criteria

Patients were included in the study only if they have type 1 diabetes mellitus on regular insulin therapy regularly visiting the Clinic.

### Exclusion criteria

Included Type 2 diabetes mellitus, hypertension, patients with malignancy, connective tissue diseases, liver dysfunction, renal dysfunction (serum creatinine > 1.2 mg/dl), congenital or acquired cardiovascular disorders, administration of drugs other than insulin (such as oral hypoglycemics, antihypertensives, antiplatelets or lipid lowering medications, aspirin, or vitamin supplements) at the time of the study and none of them was cigarette smoker.

All patients were subjected to detailed history taking, thorough clinical examination, measurement of Glycosylated Hb (HbA1c) by HPLC (high performance liquid Chromatography). Patients were considered under optimal glycemic control when their HbA1c was < 7.5% [25]. Microalbuminuria was assayed using SERA-PAK immuno-microalbumin Kit (Bayer Corporation, Benedict Ave, Tarry town, NY, USA). Persistent microalbuminuria was defined when two of three samples showed urinary albumin excretion rate of 30–300 μg/mg creatinine [26] fasting serum triglycerides, serum cholesterol using Synchron CX7 (Brea, California, USA) [27]. Serum total antioxidant capacity by colorimetric method [28], serum NO by colorimetric method [29] and Carotid intimal –media thickness (CIMT) assessment using B mode ultrasonography [30].

Measurment of serum Total Antioxidant Capacity (TAC) by colorimetric method.

The determination of antioxidative capacity was performed by the reaction of antioxidants in the sample with a defined amount of exogenously provide hydrogen peroxide (H2O2) the antioxidants in the sample eliminate a certain amount of the provided hydrogen peroxide. The residual H2O2 was determined clorometrically by an enzymatic reaction which Involves the conversion of 3,5,dichloro _2_ hydroxyl benzensulphonate to a colored product [28].

Measurement of serum Nitric oxide (NO) was done using colorimetric method. This assay determines nitric oxide concentrations based on the enzymatic conversion of nitrate to nitrite by nitrate reductase. The reaction is followed by colorimetric detection of nitrite as an azo dye product of the Griess Reaction. The Griess Reaction is based on the two-step diazotization reaction in which acidified NO2^-^ produces a nitrosating agent, which reacts with sulfanilic acid to produce the diazonium ion. This ion is then coupled to N-(1-naphthyl) ethylenediamine to form the chromophoric azo-dervative which absorbs light at 540–570 nm [29].

### Statistical analysis

Demographic and clinical data are presented as means ± SD or proportions. Differences in continuous variables between males and females were tested with the Student *t* test for normally distributed data and the Mann–Whitney *U* test for non-normally distributed data. The χ2 test for contingency tables with different degrees of freedom was obtained to detect associations between categorical independent variables. Adjustment for multiple confounding was done using linear regression analysis with a manual backward procedure. Multivariate analyses were preceded by estimation of the correlation between potential confounders. A significance level of < 0.05 was used. All statistical analysis was done using the SPSS software package for Windows, version 15.0 (SPSS, Chicago, IL).

## Results

**Figure 1:**
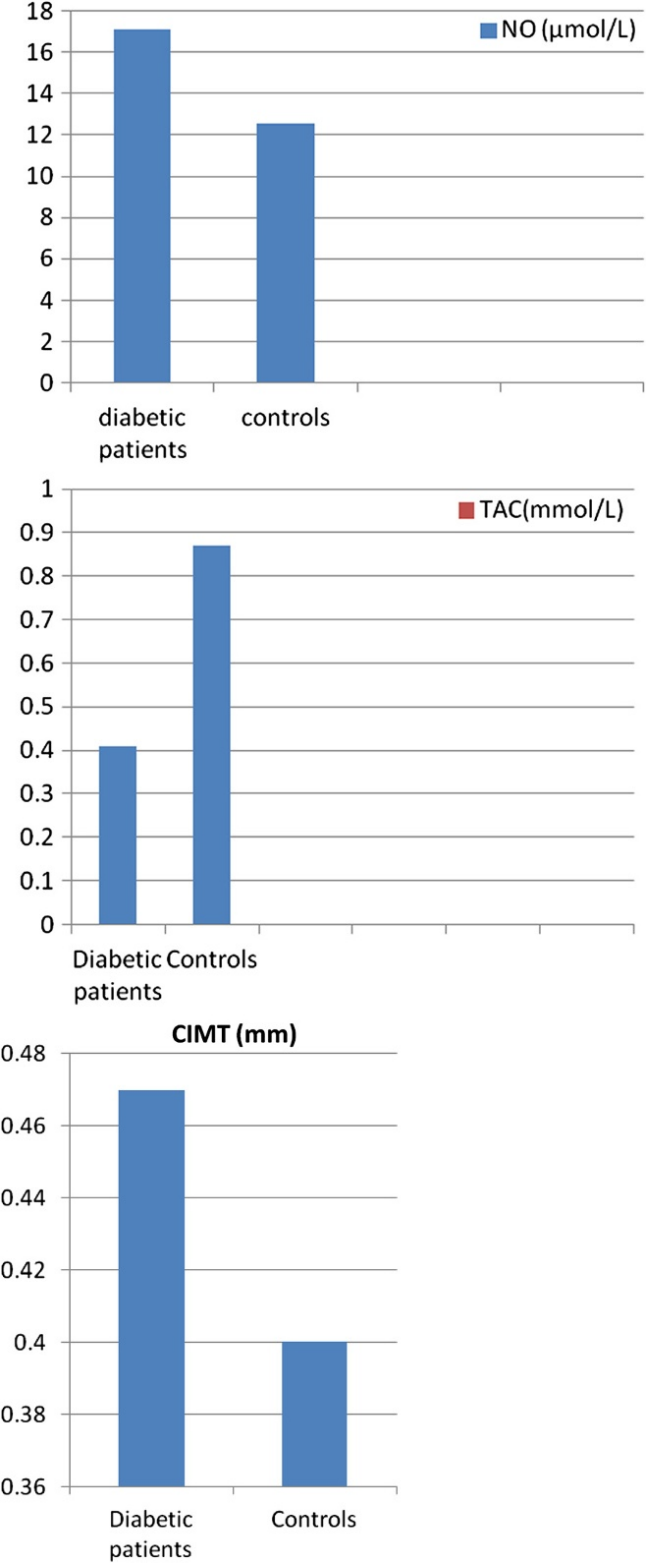
**Comparison between patients and controls regarding values of nitric oxide and total antioxidant capacity.**

- Patients mean systolic blood pressure (SBP) was103.500 ± 18.077 mmHg and mean diastolic blood pressure (DBP) was 68 ± 10.302 mmHg. Three (6%) of our patients had SBP and DBP > 95% percentile while 97% were within the normal ranges.
- Diabetic patients showed significant increase in nitric oxide and decrease in total antioxidant capacity level than controls (P value < 0.001)] (Table 1), Figure 1.

- NO level was higher in patients with suboptimal glycemic control than those with optimal glycemic control [(23.38 ± 6.7 μmol/L) vs (16.33 ± 5.6 μmol/L)] (P value = 0.017) and in patients with nephropathy than patients without nephropathy [(22.00 ± 4.14 μmol/L) vs (16.67 ± 6.5 μmol/L)] (P value = 0.033).
- No statistically significant difference was found in TAC level between diabetic patients with and without nephropathy or between patients with suboptimal and optimal glycemic control (p value >0.05)
- Serum total cholesterol and triglycerides were significantly higher in studied patients than control group, although within normal range.
- Carotid intima media thickness was significantly increased in diabetic patients compared to normal controls [(mean 0.46 ± 0.04 mm) vs (mean 0.39 ±0.02 mm)] (P < 0.001) (Table 1), Figure 1.
- Carotid intima media thickness was significantly increased in diabetic patients with suboptimal than those with optimal glycemic control [(0.46 ± 0.04 cm) vs (0.40 ± 0.02 cm)] (P value 0.04), in patients with diabetes duration > 5 years than those with diabetes duration < 5 years [(0.50 ± 0.03 mm) vs (0.43 ± 0.02 mm)] (P value <0.001)] (Table 2) and in diabetic patients with than patients without nephropathy [ (0.49 ± 0.02) vs (0.40 ± 0.04)] (P value = 0.009).
- A statistically significant positive correlation was found between carotid intima media thickness and age, duration of diabetes mellitus, systolic and diastolic blood pressure and nitric oxide (P value < 0.001 and <0.001, 0.002, 0.007 and 0.01 respectively) and a significant negative correlation was found between carotid intima media thickness and total antioxidant capacity (P value = 0.02) (Table 3). No significant correlation was found between carotid intima media thickness and mean HbA1c, cholesterol or triglycerides (P value >0.05).

**Table 1:** **Comparison between diabetic patients and control group regarding studied parameters**

Groups			t-test	
	Patients(n50) Mean ± SD	Controls(n50) Mean ± SD	t	P-value
Age (years)	9.78 ± 3.45	9.86 ± 3.14	-0.12	0.90
BMI(kg/m2)	20.120 ± 4.155	18.740 ± 3.87	t = 1.72	0.09
Cholesterol (mg/dl)	147.35 ± 38.92	120.68 ± 35.26	t = 3.5	0.0005*
Triglycerides (mg/dl)	80.86 ± 25.10	62.40 ± 20.50	t = 4.03	0.0001*
NO (μmol/L)	17.07 ± 6.36	12.57 ± 4.74	3.98	<0.001*
TAC (mmol/L)	0.41 ± 0.29	0.87 ± 0.23	-8.87	<0.001*
CIMT (mm)	0.47 ± 0.04	0.40 ± 0.02	9.66	<0.001*
Gender	37/13	36/14	0.437#	0.509

*NO* nitric oxide, *TAC* Total antioxidant capacity, *CIMT* carotid intima media thickness; #= Chi-Square.

*Indicate statistical significance.

**Table 2:** **Comparison between patients with diabetes duration <5 years and >5 years regarding studied parameters**

	Patients with diabetes duration ≤ 5 years(n = 28)	Patients with diabetes duration > 5 years(n = 22)	T-test	
	Mean ± SD	Mean ± SD	t	P-value
Age (years)	7.64 ± 2.80	12.50 ± 1.946	-6.92	<0.001*
Mean HbA_1_c%	8.47 ± 1.98	9.73 ± 2.07	-2.19	0.03*
Cholesterol (mg/dl)	134.36 ± 38.34	162.50 ± 35.23	-1.94	0.07
Triglycerides (mg/dl)	79.13 ± 24.74	81.86 ± 25.54	0.38	0.7
NO (μmol/L)	16.27 ± 6.70	18.09 ± 5.89	-1.01	0.32
TAC (mmol/L)	0.45 ± 0.31	0.36 ± 0.25	1.18	0.25
CIMT (mm)	0.44 ± 0.03	0.50 ± 0.03	-7.09	<0.001*

*NO* nitric oxide, *TAC* Total antioxidant capacity, *CIMT* carotid intima media thickness.

*Indicate statistical significance.

**Table 3:** **Correlation between CIMT, serum NO and serum TAC in diabetic patients**

		CIMT (mm)	NO (μmol/L)
Age (years)	r	0.741	
	P-value	<0.001*	
Duration of diabetes (years)	r	0.656	
	P-value	<0.001*	
SBP (mmHg)	r	0.126	
	P-value	0.002*	
DBP (mmHg)	r	0.379	
	P-value	0.007*	
NO (μmol/L)	r	0.40	
	P-value	0.01*	
TAC(mmol/L)	r	-0.34	-0.53
	P-value	0.02*	<0.001*

*CIMT* carotid intima media thickness, *SBP* systolic blood pressure, *DBP* diastolic blood pressure, *NO* nitric oxide, *TAC* Total antioxidant capacity.

*Indicate statistical significance.

## Discussion

The present study showed a significant increase in serum nitric oxide (NO) level in diabetic patients than controls which came in agreement with many studies [31–33]. Pitocco et al. [34] suggested a reduced asymmetric dimethyl arginine (ADMA) inhibition of NOS as possible mechanism involved in the pathogenesis of oxidative stress in female subjects with a short duration and uncomplicated type 1 diabetes.

Serum NO level was significantly increased in diabetic patients with than those without nephropathy which came in accordance to previous studies [35, 36]. Prabhakar, [37] reported that the enhanced NO production may contribute to hyperfiltration and microalbuminuia at early diabetic nephropathy. NO level was significantly higher in patients with suboptimal than optimal glycemic control which came in agreement with others [38].

A significant decrease in total antioxidant capacity level was found in patients than controls which came in agreement with several studies [39–42]. Lack of difference in TAC level between our studied patients with and without nephropathy is concordant with El-desoky et al. [43] who found that plasma TAC in patients of diabetes mellitus type I with nephropathy was not significantly decreased as compared with diabetes mellitus type 1 with no nephropathy and they concluded that TAC cannot be used to differentiate between diabetes mellitus type 1 with and without nephropathy.

Serum total cholesterol and triglycerides were significantly higher in studied diabetic patients compared to control group although within the normal range. This agreed with Margeisdottir et al. [44] who attributed those findings to the young age of the patients.

Diabetic patients showed a significant increase in CIMT mean values compared to controls. Those results came in accordance to several studies [45–47]. A significant increase in CIMT in patients with nephropathy compared to patients without nephropathy came in concordance with Gul et al. [47] suggesting that diabetic microangiopathy is related with macroangiopathy and in patients with suboptimal glycemic control compared to patients with optimal glycemic control which came in concordance with Abdelghaffar et al. [46].

CIMT was directly correlated with age in studied diabetic patients which agreed with many studies [46–48]. A strong direct correlation was found between mean CIMT and patients systolic and diastolic blood pressure which agreed with several studies [46, 48, 49]. The relationship between increased CIMT and blood pressure suggests that smooth muscle proliferation plays a role in the early diffuse thickening of the arterial wall [49].

No significant correlation was found between mean CIMT and metabolic control parameter HbA1c, this agreed with many studies [47, 50]. Data from the literature indicated that, in contrast to the functional impairment of the endothelium, structural changes are not correlated to single parameter such as the HbA1c at a young age [50].

A statistically significant positive correlation was found between carotid intima media thickness and nitric oxide level which agreed with Dursun et al. [51] who explored the relation between CIMT and oxidative stress markers in type 2 diabetic patients on maintenance hemodialysis. Zineh et al. [52] reported the association between NOS3 polymorphisms and arterial stiffness in children with type 1 diabetes. Insulin acts by modulating the release of vasodilator substances, such as nitric oxide and prostaglandins, from vascular endothelium, by both stimulating and inhibiting the sympathetic nervous system and by protecting smooth muscle cells in blood vessel from apoptosis induced by oxidative stress [53]. Thus the vasodilatory and antioxidant effects of insulin are depressed in case of insulin deficiency (i.e. type 1 diabetes) [54].

A negative correlation was found between CIMT and total antioxidant capacity level. Lower serum TAC levels were observed in atherosclerotic coronary artery disease patients with an inverse association with number of damaged coronary vessels [55]. Other research reported increased DNA damage in the nucleus of coronary cells and decreased plasma TAC level in coronary artery disease patients [56].

## Conclusion

The significant elevation in nitric oxide and reduction in total antioxidant capacity in children and adolescents with type 1 diabetes mellitus together with their correlation with carotid intima media thickness may reflect the role of oxidative stress in the development of atherosclerosis in young type 1 diabetic subject. Further studies to measure other antioxidant markers like glutathione or antioxidants or markers of oxidative stress like malondialdehyde in relation to carotid intima media thickness are warranted.

## Authors’ original submitted files for images

Below are the links to the authors’ original submitted files for images. Authors’ original file for figure 1

## Abbreviations

CIMT: Carotid intima media thickness
DM: Diabetes mellitus
HbA1c: Glycosylated hemoglobin
NO: Nitric oxide
TAC: Total antioxidant capacity
SBP: Systolic blood pressure
DBP: Diastolic blood pressure
ELISA: Enzyme linked immune sorbent assay.
