# Supplementary figures and images for: Relation between carotid intima media thickness and oxidative stress markers in type 1 diabetic children and adolescents
Source: J Diabetes Metab Disord. 2013 Dec 19;12:50. doi: 10.1186/2251-6581-12-50 (PMC7968341; doi:10.1186/2251-6581-12-50)

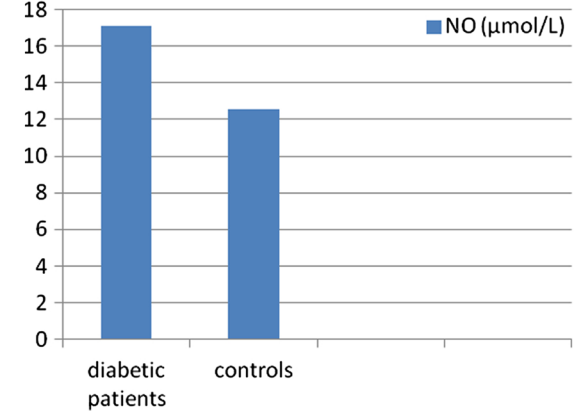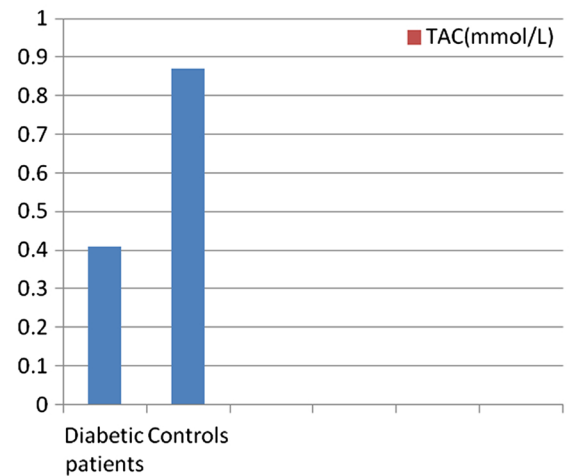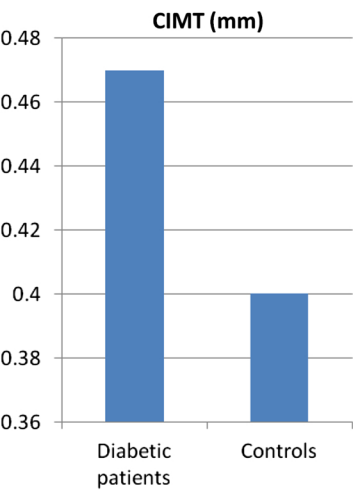

Supplement: Supplementary file 1 — Authors’ original file for figure 1 [file 40200_2013_182_MOESM1_ESM.pdf]
